# Data of fluorescence, UV–vis absorption and FTIR spectra for the study of interaction between two food colourants and BSA

**DOI:** 10.1016/j.dib.2016.06.025

**Published:** 2016-06-23

**Authors:** Tian Li, Zhengjun Cheng, Lijun Cao, Xiaohui Jiang, Lei Fan

**Affiliations:** a Chemical Synthesis and Pollution Control Key Laboratory of Sichuan Province, China West Normal University, Nanchong 637002, PR China; b School of Chemistry and Chemical Engineering, Yangzhou University, Yangzhou, 225002, PR China

**Keywords:** Bovine serum albumin, Acid red 1, Acid green 50, Data

## Abstract

In this data article, the fluorescence, UV–vis absorption and FTIR spectra data of BSA-AR1/AG50 system were presented, which were used for obtaining the binding characterization (such as binding constant, binding distance, binding site, thermodynamics, and structural stability of protein) between BSA and AR1/AG50.

**Specifications Table**

**Table:** 

Subject area	Chemistry
More specific subject area	Analytical chemistry
Type of data	Table, figure
How data was acquired	Cary Eclipse fluorescence spectrofluorimeter (Varian, USA), UV-3600 spectrophotometer (Shimadzu, Japan), Nicolet-6700 FTIR spectrometer (Thermoscientific, USA)
Data format	Raw, analyzed
Experimental factors	The solution of BSA was prepared in phosphate-buffer (0.05 M NaH_2_PO_4_-Na_2_HPO_4_, pH=4.8, 5.5, 6.3 and 7.4) without/with salt (NaCl, 99.5% purity) or ethanol (99.7% purity)
Experimental features	Fluorescence, UV–vis absorption and FTIR spectra were employed to investigate binding characterization of BSA with AR1/AG50 at different conditions
Data source location	Nanchong, China
Data accessibility	Data are provided with this article

**Value of the data**

• The data are intuitionistic for readers to compare the binding affinity of AR1/AG50 with BSA;
• The data are helpful to readers for understanding further the related parameters calculated;
• The data may be of great help to study in detail the similar systems.

## 1 Data

The interaction data of BSA with AR1/AG50 were determined using Cary Eclipse fluorescence spectrofluorimeter (Varian, USA), UV-3600 spectrophotometer (Shimadzu, Japan) or Nicolet-6700 FTIR spectrometer; and these data were shown as fluorescence quenching spectra, the Stern–Volmer plots in the absence and presence of ethanol/NaCl, RLS spectra, UV–vis absorption spectra, UV-melting profiles, synchronous fluorescence spectra, and FTIR spectra. Corresponding parameters were calculated based on the interaction data.

**Fig. 1:**
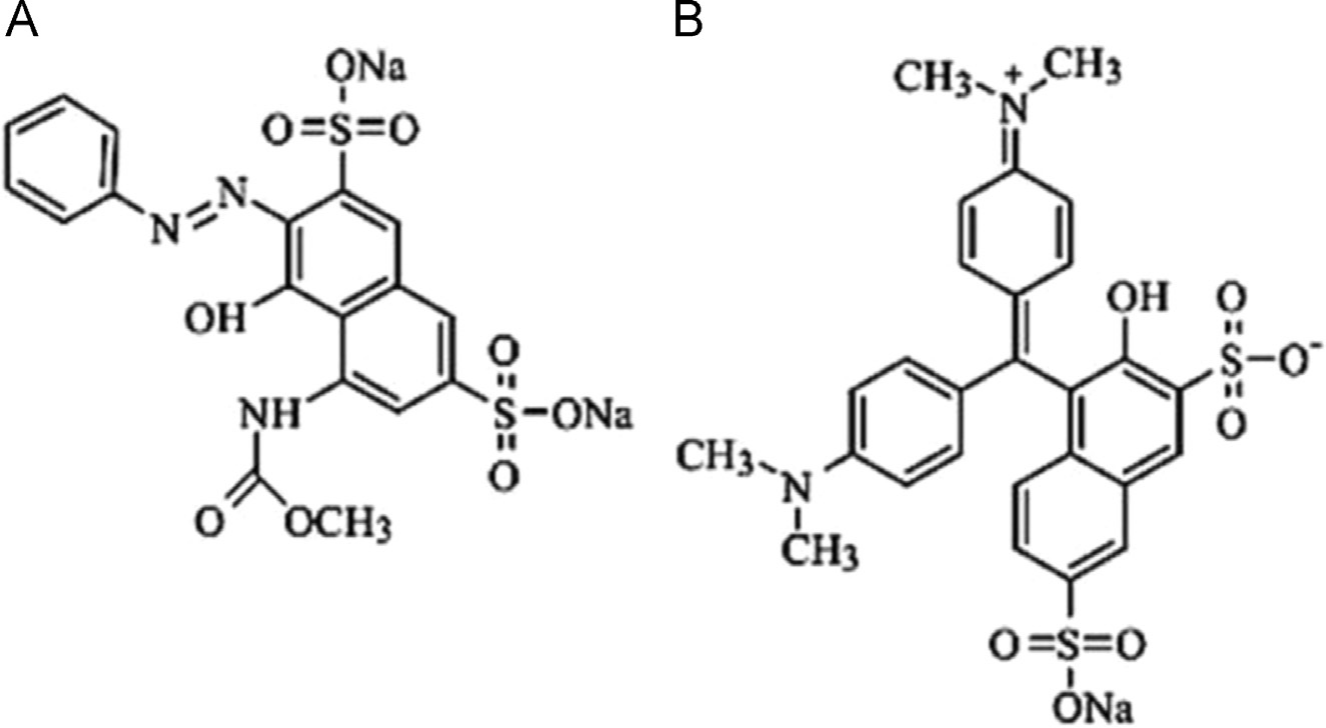
The structures of acid red1 (A) and acid green 50 (B).

In addition, to make the figures in the text become clearer, all of the figures were processed by Photoshop 8.1 software. ChemOffice 2008 was used for drawing the structures of acid red 1 and acid green 50 (Fig. 1).

## 2 Experimental design, materials and methods

### 2.1 Materials

Bovine Serum Albumin (BSA, Fraction V, 98% purity, *M_r_*= 68,000 g/mol, CAS: 9048-46-8) was purchased from *Ruibio Company*. AR1 (CAS: 3734-67-6) and AG50 (CAS: 3087-16-9) were obtained from *J&K Scientific Ltd.* (Beijing, China) and *Acros Organics* (New Jersey, USA), respectively. And all other chemicals were analytical reagent grade.

### 2.2 Methods

#### 2.2.1 Fluorescence quenching of BSA by AR1/AG50

3.0 mL BSA solution (2.0 μM) was titrated by successive additions of AR1/AG50 solution with the concentration of 3.0×10^−4^ mol L^−1^ at different conditions (pH=4.8, 5.5, 6.3 or 7.4, *T*=293, 298, 304 or 310 °K, *c*(NaCl)=0.0, 0.04, 0.09 or 0.15 M, and/or ethanol content (%)=0%, 5% or 10%), and the final concentration of AR1/AG50 was kept at 11.54×10^−6^ mol L^−1^. The fluorescence quenching of BSA with the addition of AR1/AG50 was recorded in the range of 300–500 nm by Cary Eclipse fluorescence spectrofluorimeter (Varian, USA). The width of the excitation and emission slit was adjusted at 5 nm, and the excitation wavelength was selected at 280 nm. The temperature of samples was kept by recycle water during the whole experiment. All fluorescence titration experiments were done manually by 50 µL microsyringe.

**Fig. 2:**
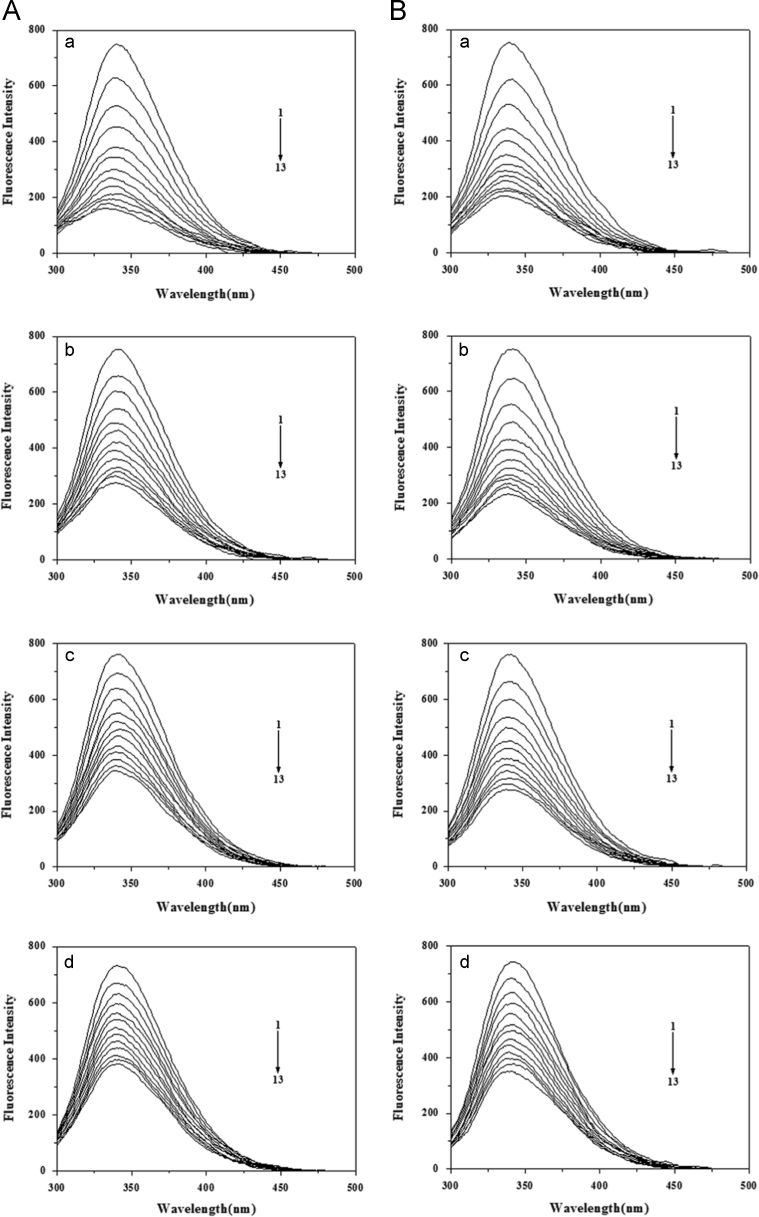
Fluorescence quenching spectra of BSA without salt by AR1 (A) or AG50 (B) at pH 4.8 (a), 5.5 (b), 6.3 (c) and 7.4 (d), respectively. *λ*_ex_=280 nm; *c*(BSA)=2.0 μM; *c*(AR1)=*c*(AG50) 1–13=0, 1.00, 1.99, 2.97, 3.95, 4.92, 5.88, 6.84, 7.79, 8.74, 9.68, 10.61, 11.54 μM; *T*=298 °K.

The figures of fluorescence quenching spectra (Fig. 2) were made using Origin 7.5.

#### 2.2.2 UV–vis absorption spectra of BSA, AR1 and AG50

**Fig. 3:**
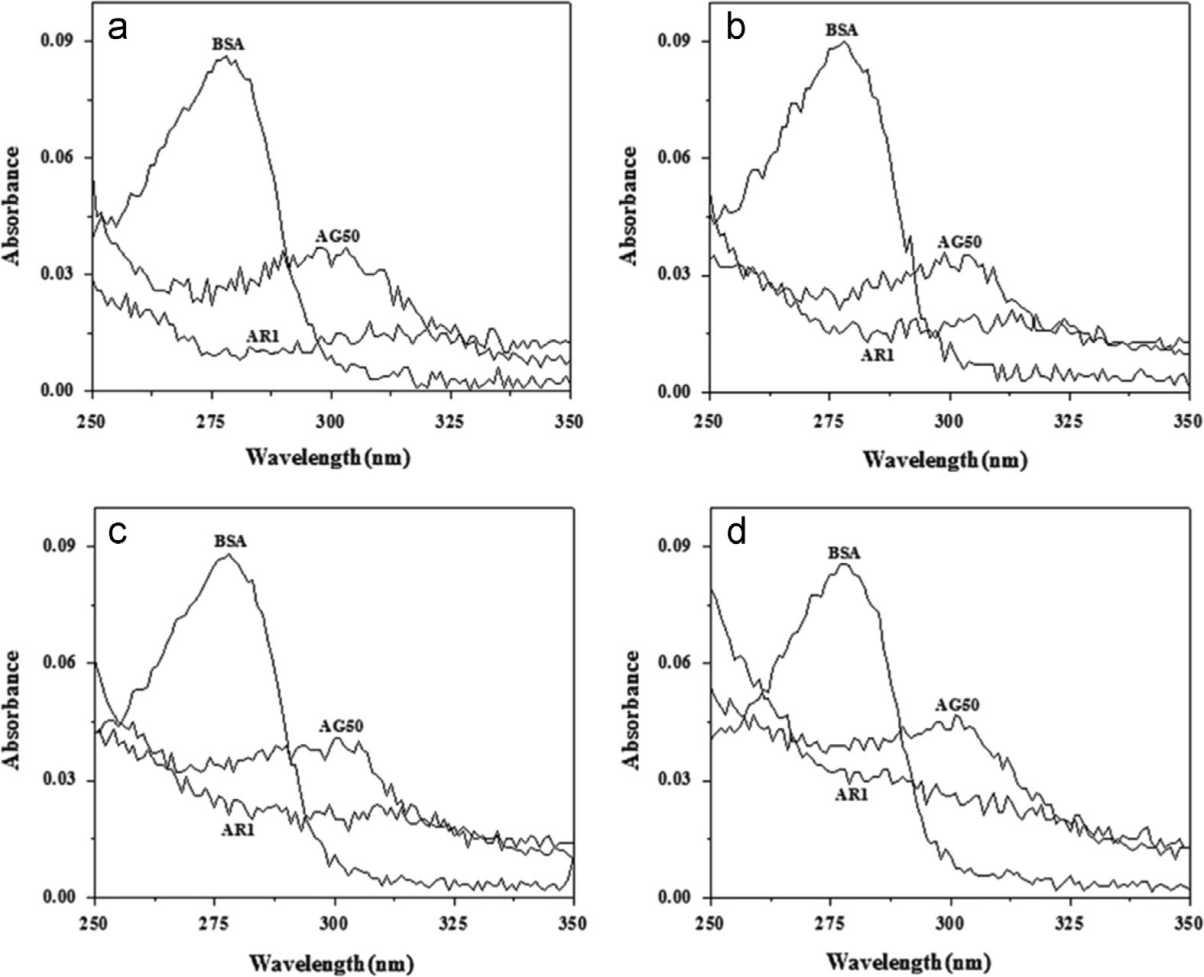
Absorption spectra of BSA, AR1 and AG50 at pH 4.8 (a), 5.5 (b), 6.3 (c) and 7.4 (d); *c*(BSA)=2.0 μM; *c*(AR1)=*c*(AG50)=1.99 μM; *T* =298 °K.

Absorption spectra of AR1 (1.99 μM), AG50 (1.99 μM) and BSA (2.0 μM) in 3.0 mL phosphate-buffer was determined by UV-3600 spectrophotometer (Shimadzu, Japan) in the range of 250–350 nm, respectively; and corresponding figures (Fig. 3) were done by Origin 7.5.

#### 2.2.3 Spectral overlap of BSA with AR1/AG50

The fluorescence emission spectra of BSA (2.0 μM) at pH=4.8, 5.5, 6.3 or 7.4 and [NaCl]=0, 0.04, 0.09 or 0.15 M were carried out by Cary Eclipse fluorescence spectrofluorimeter, respectively. Other scanning parameters were the same as those of the fluorescence titration experiments. The UV–vis absorption spectra of AR1/AG50 (1.99 μM) were determined on UV-3600 spectrophotometer at pH=4.8, 5.5, 6.3 or 7.4 and [NaCl]=0, 0.04, 0.09 or 0.15 M, respectively. Fluorescence emission and UV–vis absorption spectra were recorded in the range of 250–500 nm.

**Fig. 4:**
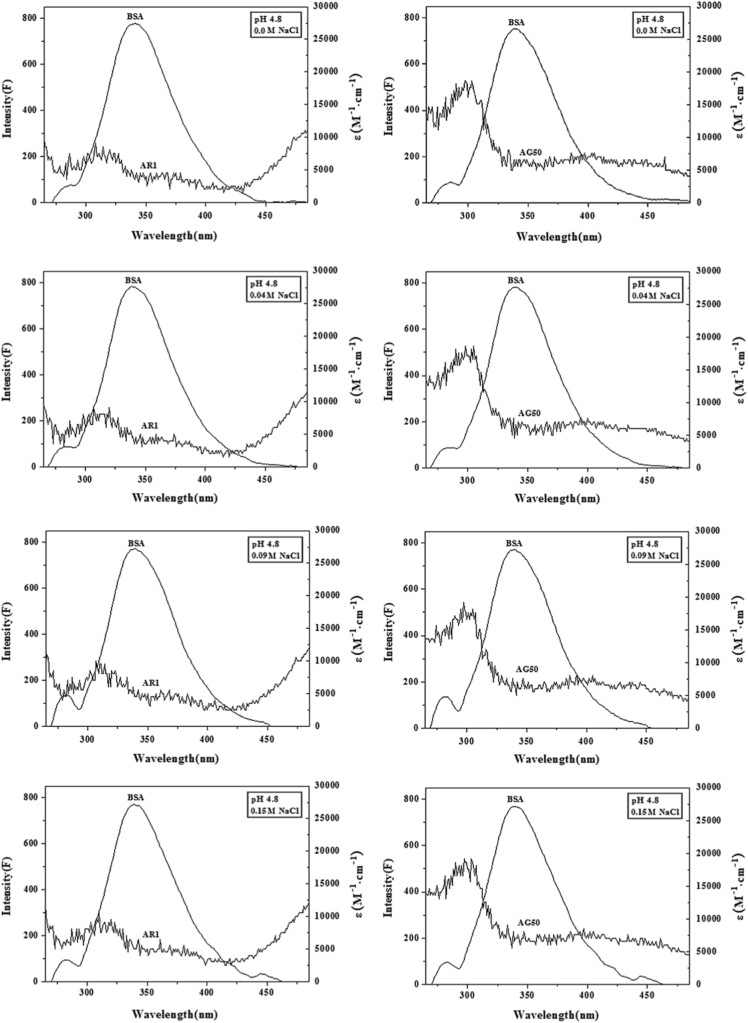

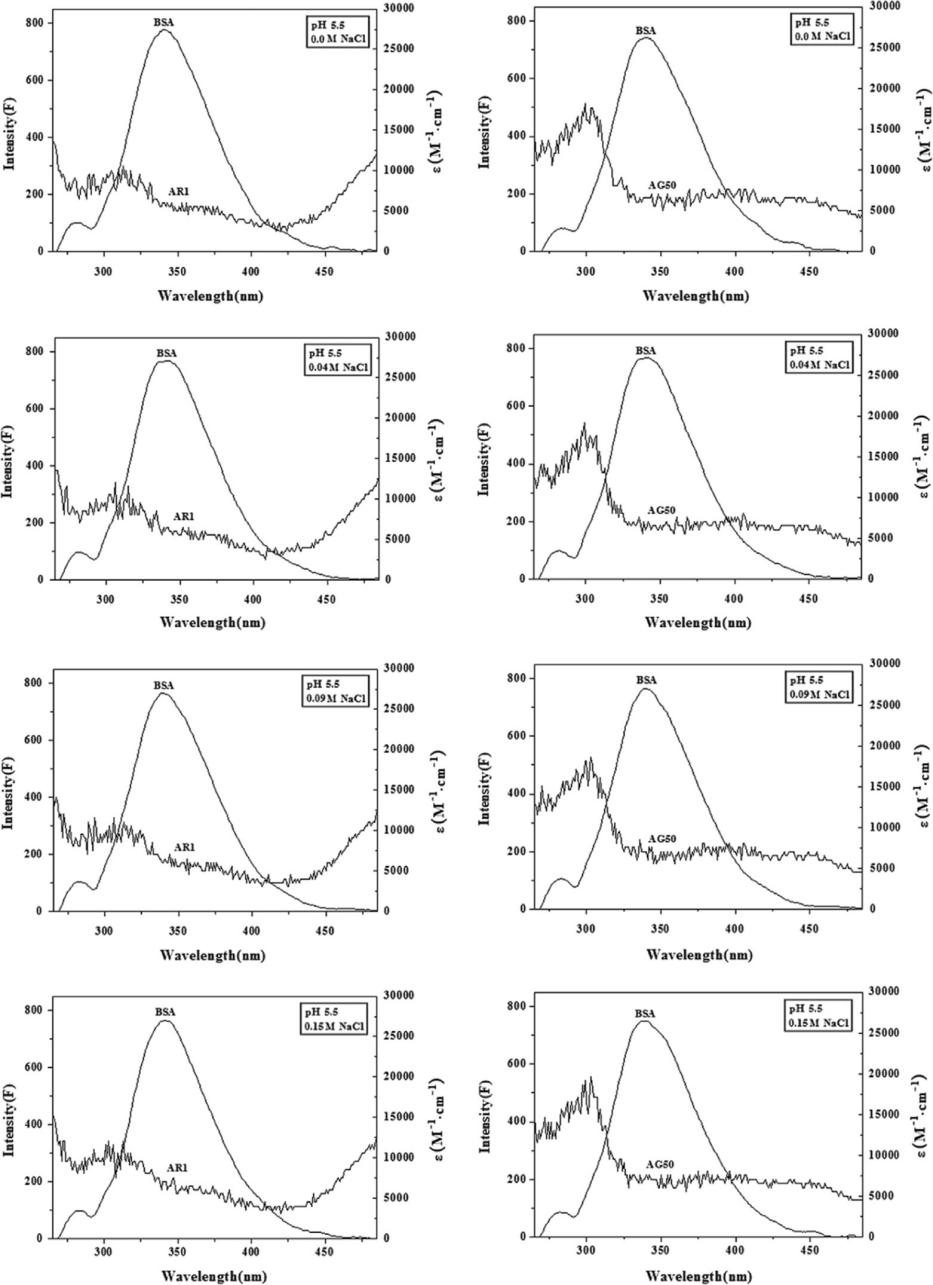

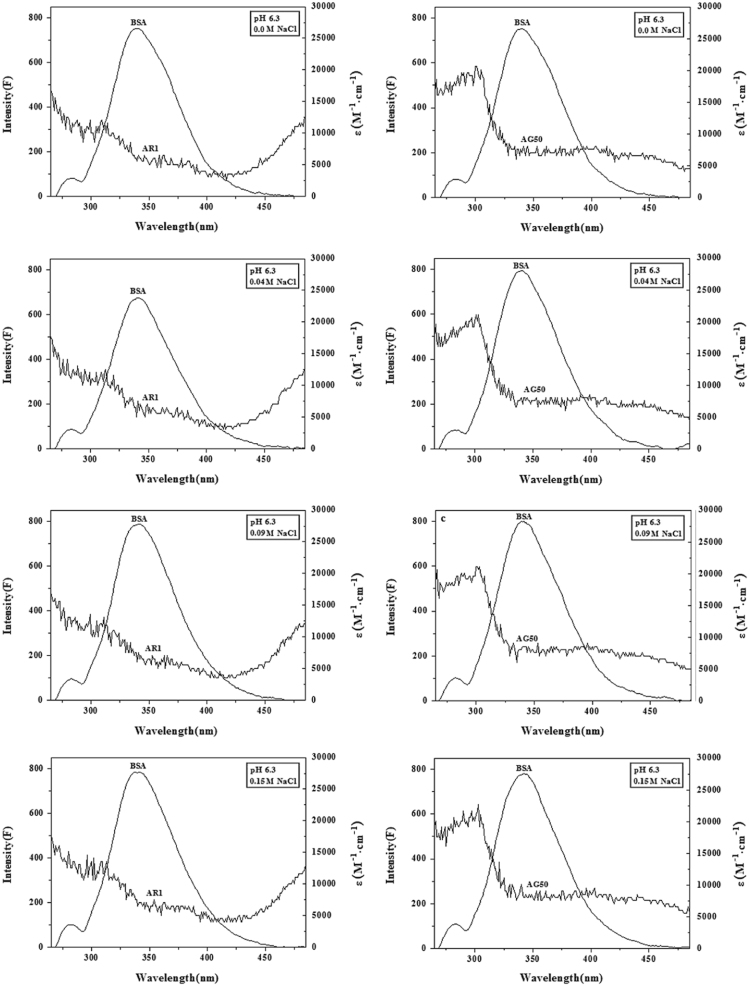

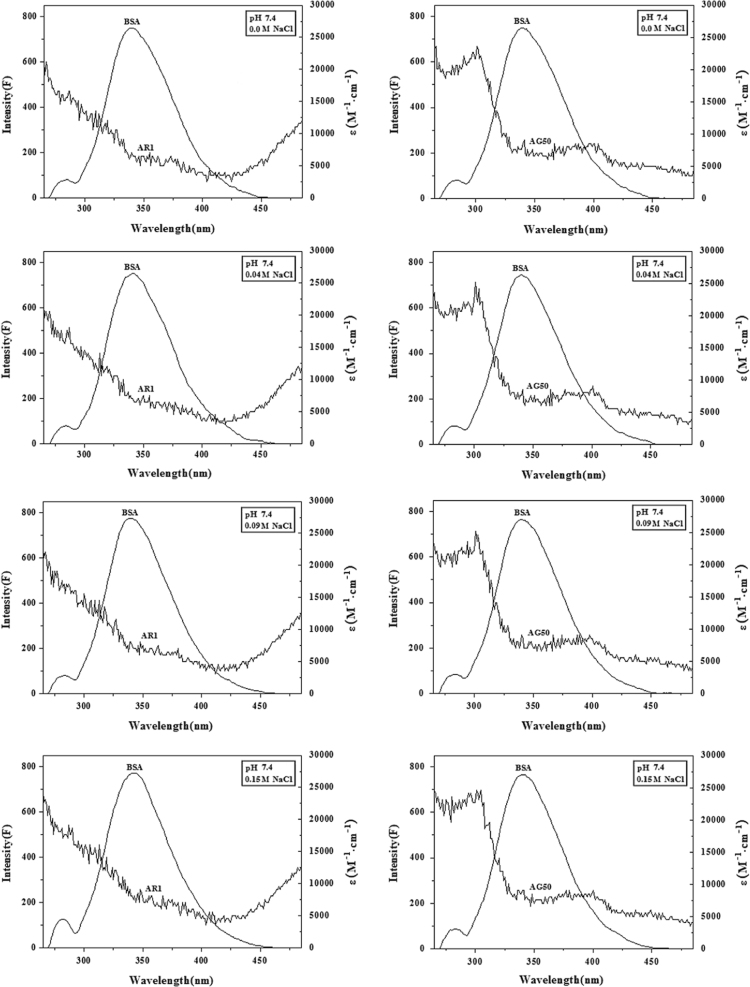
Spectral overlap of UV–vis absorption spectrum of AR1 or AG50 with the fluorescence emission spectrum of BSA at pH 4.8, 5.5, 6.3 or 7.4; *c*(BSA)=2.0 μM, *c*(AR1)=*c*(AG50)=1.99 μM, *T*=298 °K.

The figures of spectral overlap between BSA and AR1/AG50 (Fig. 4) were done by Origin 7.5.

#### 2.2.4 The S–V plots of BSA-AR1/AG50 system

**Fig. 5:**
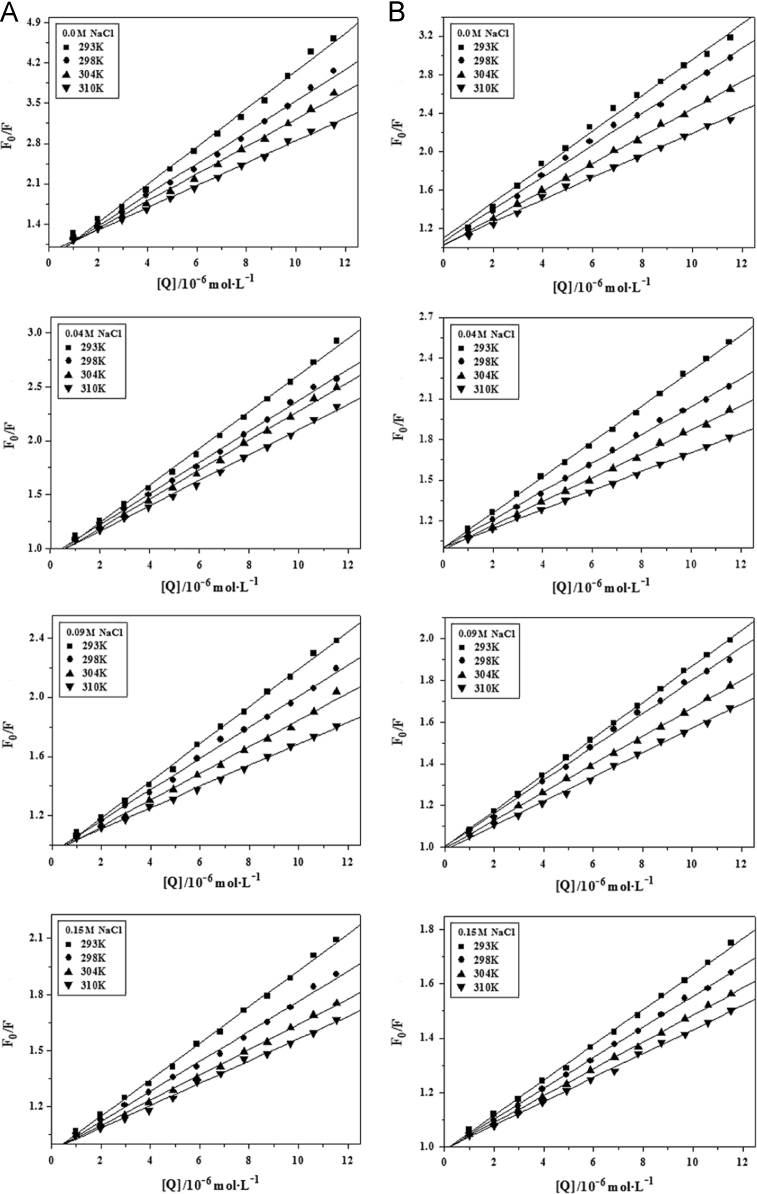

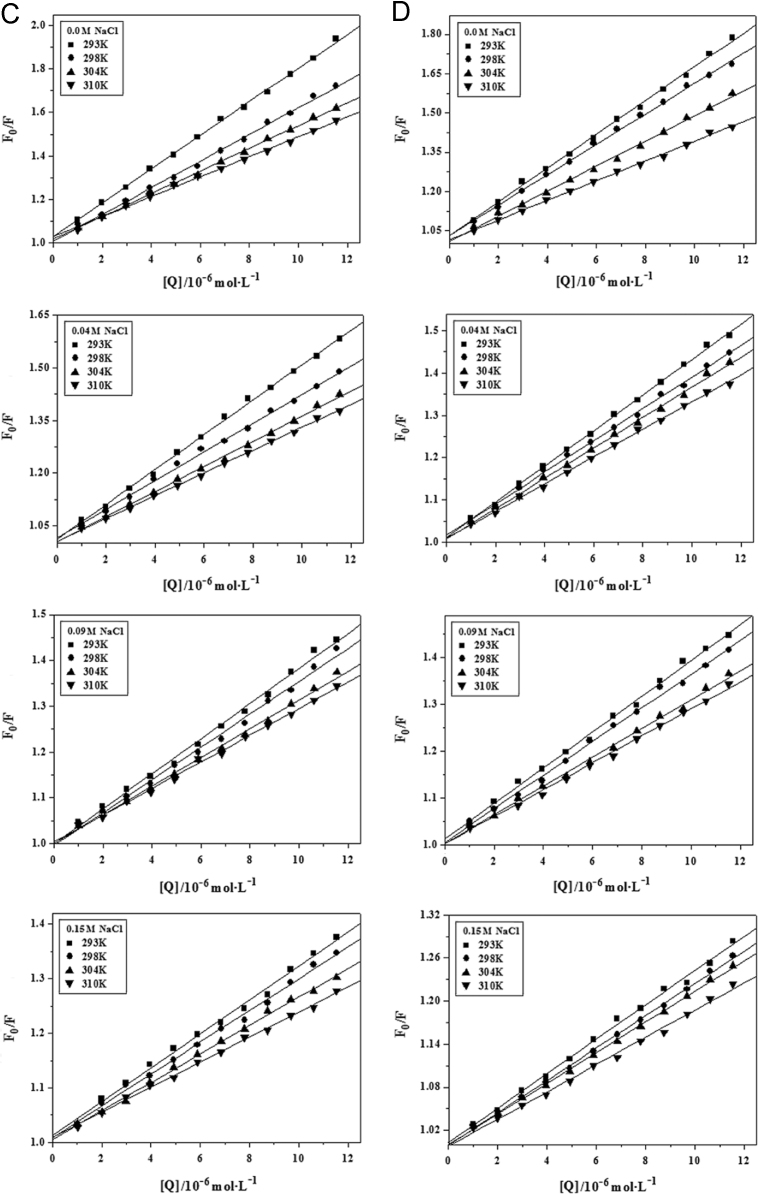
The S–V plots for the quenching of BSA by AR1 (A and C) or AG50 (B and D) at pH=4.8 (A and B) and 7.4 (C and D); *λ*_ex_=280 nm.

The measured fluorescence quenching data of BSA by AR1/AG50 at different conditions were corrected [1] and fitted by Origin 7.5 based on Eq. (1) (Fig. 5), and corresponding values were listed in Table 1.

(1) $$\frac{F_{0}}{F}={\left(\frac{F_{0}}{F}\right)}_{m}\eta =1+K_{\mathrm{SV}}[Q]$$

#### 2.2.5 RLS spectra of BSA-AR1/AG50 system

**Fig. 6:**
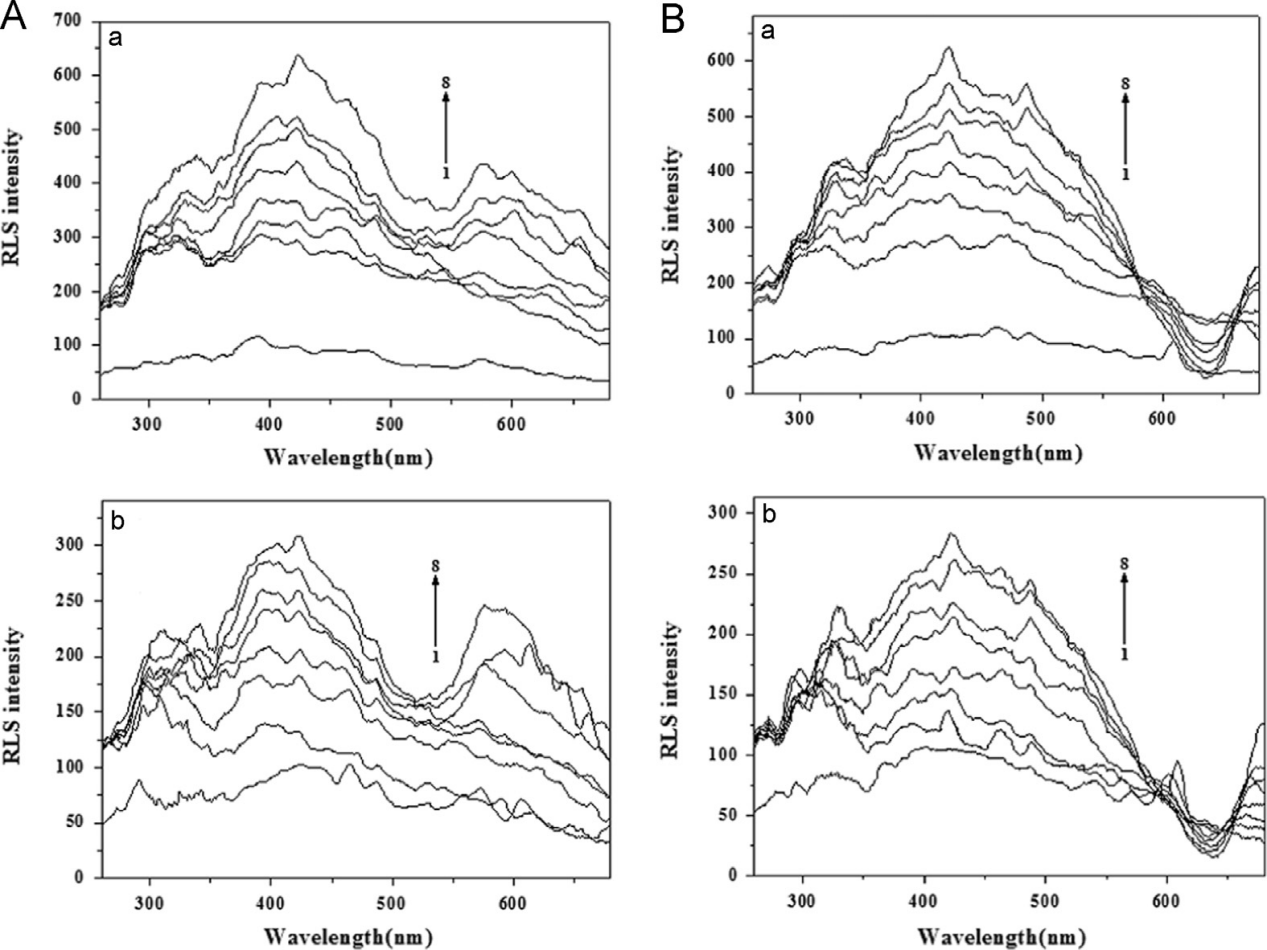
RLS spectra of the BSA-AR1 (A) and BSA-AG50 (B) systems at pH 4.8 (a) and 7.4 (b). Curve 1: only AR1 or AG50 (1.99 μM); curve 2: only BSA (2.0 μM); *c*(AR1)=*c*(AG50) 3–8=1.99, 3.95, 5.88, 7.79, 9.68, 11.54 μM.

RLS spectra of BSA (2.0 μM) with the addition of AR1/AG50 (0-11.54 μM) at Δ*λ*=0 nm were determined in the range of 250–700 nm by Cary Eclipse fluorescence spectrofluorimeter at pH 4.8 and 7.4, respectively; and corresponding figures of RLS spectra (Fig. 6) were done by Origin 7.5. Other scanning parameters were the same as those of the fluorescence titration experiments.

#### 2.2.6 UV–vis absorption spectra of BSA-AR1/AG50 system

**Fig. 7:**
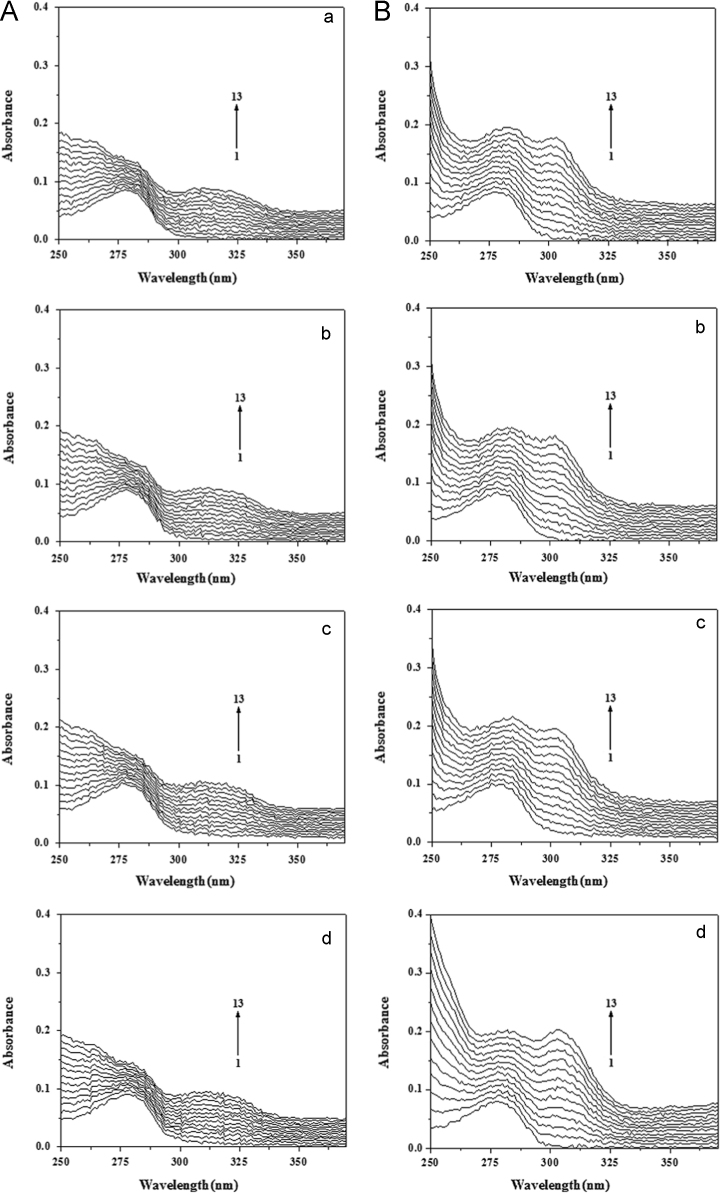
UV–vis absorption spectra of BSA in the presence of AR1 (A) or AG50 (B) at pH 4.8 (a), 5.5 (b), 6.3 (c) and 7.4 (b); *c*(BSA)=2.0 μM; *c*(AR1)=*c*(AG50) 1–13=0, 1.00, 1.99, 2.97, 3.95, 4.92, 5.88, 6.84, 7.79, 8.74, 9.68, 10.61, 11.54 μM; *T*=298 °K.

UV–vis absorption spectra (in the range of 250–400 nm) of BSA (2.0 μM) in the presence of AR1/AG50 (0–11.54 μM) at pH 4.8, 5.5, 6.3 or 7.4 were determined on UV-3600 spectrophotometer, and their figures (Fig. 7) were made by Origin 7.5.

#### 2.2.7 The effect of ethanol on the quenching plots of BSA-AR1/AG50 system

**Fig. 8:**
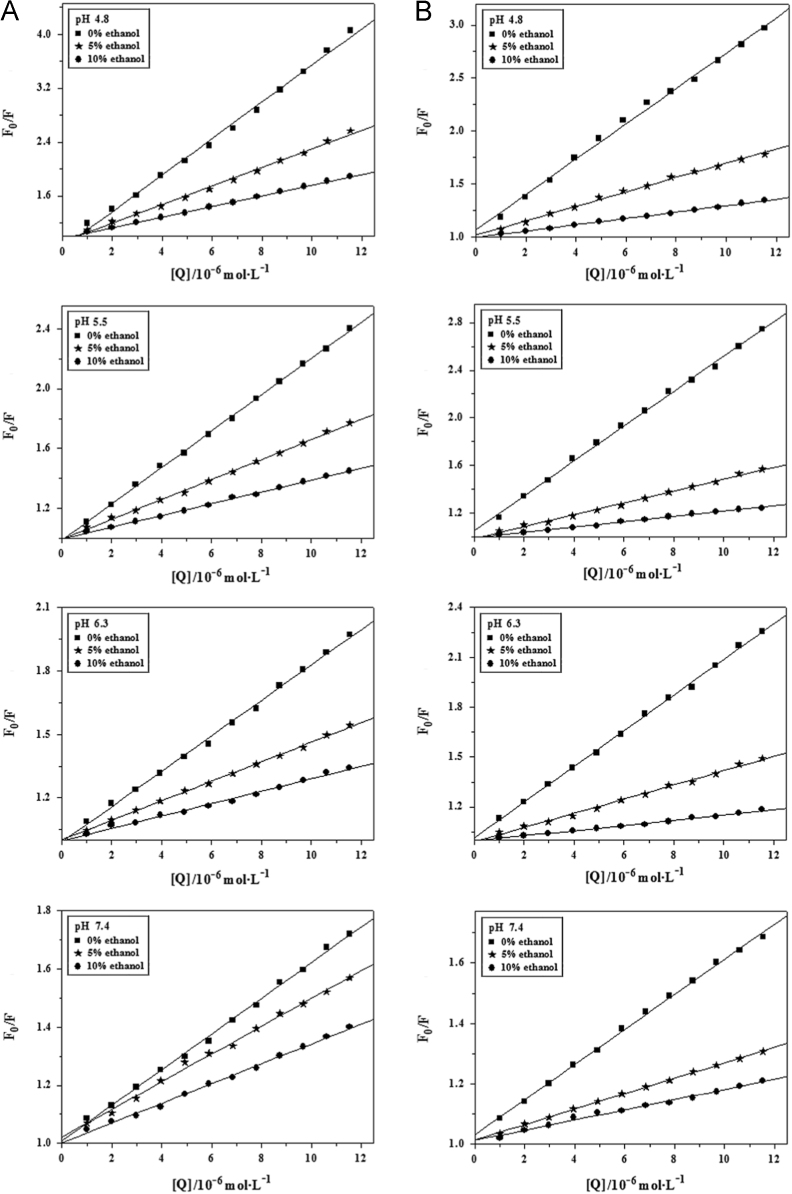
Effects of increasing ethanol content on the quenching plots of BSA by AR1 (A) or AG50 (B) at different pH values, *T*=298 °K, *λ*_ex_=280 nm.

The measured fluorescence quenching data of BSA by AR1/AG50 without or with ethanol content (5% or 10%) at pH=4.8, 5.5, 6.3 and 7.4 were corrected [1] and fitted by Origin 7.5 based on Eq. (1) (Fig. 8).

#### 2.2.8 The effect of NaCl on the absorption spectra of BSA-AR1/AG50 system

**Fig. 9:**
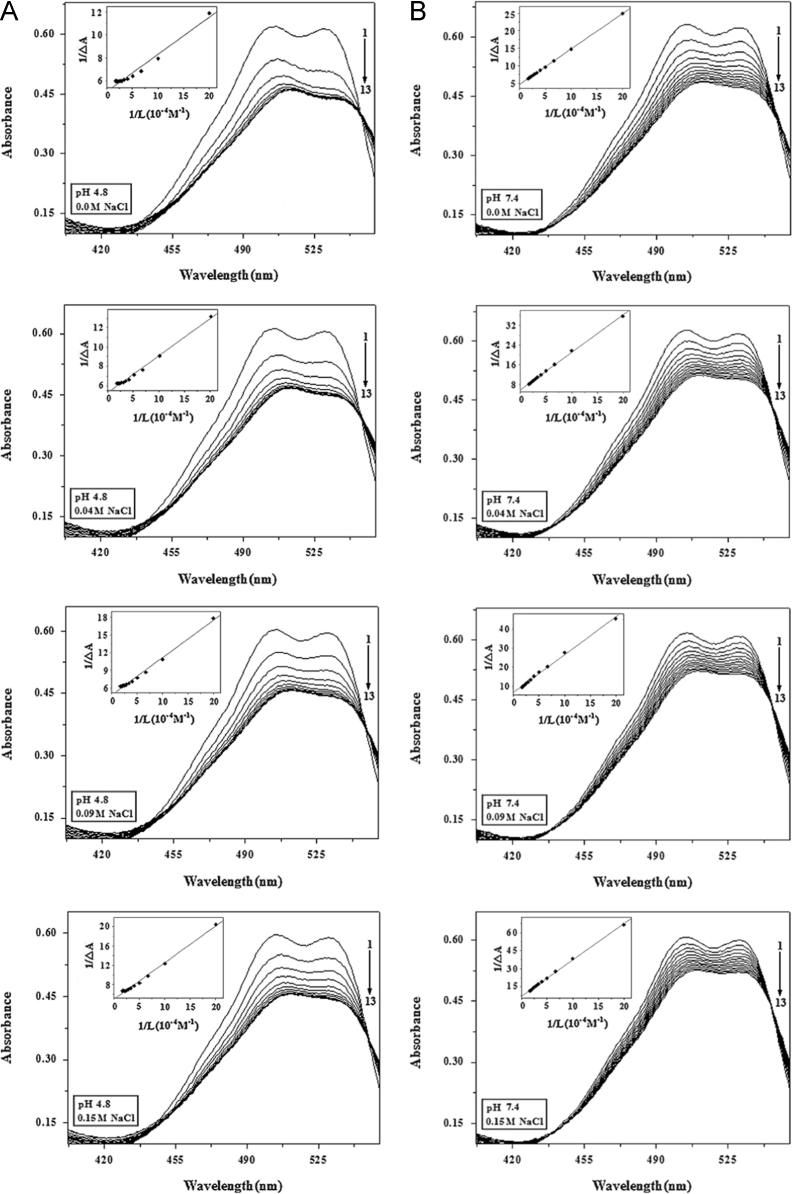

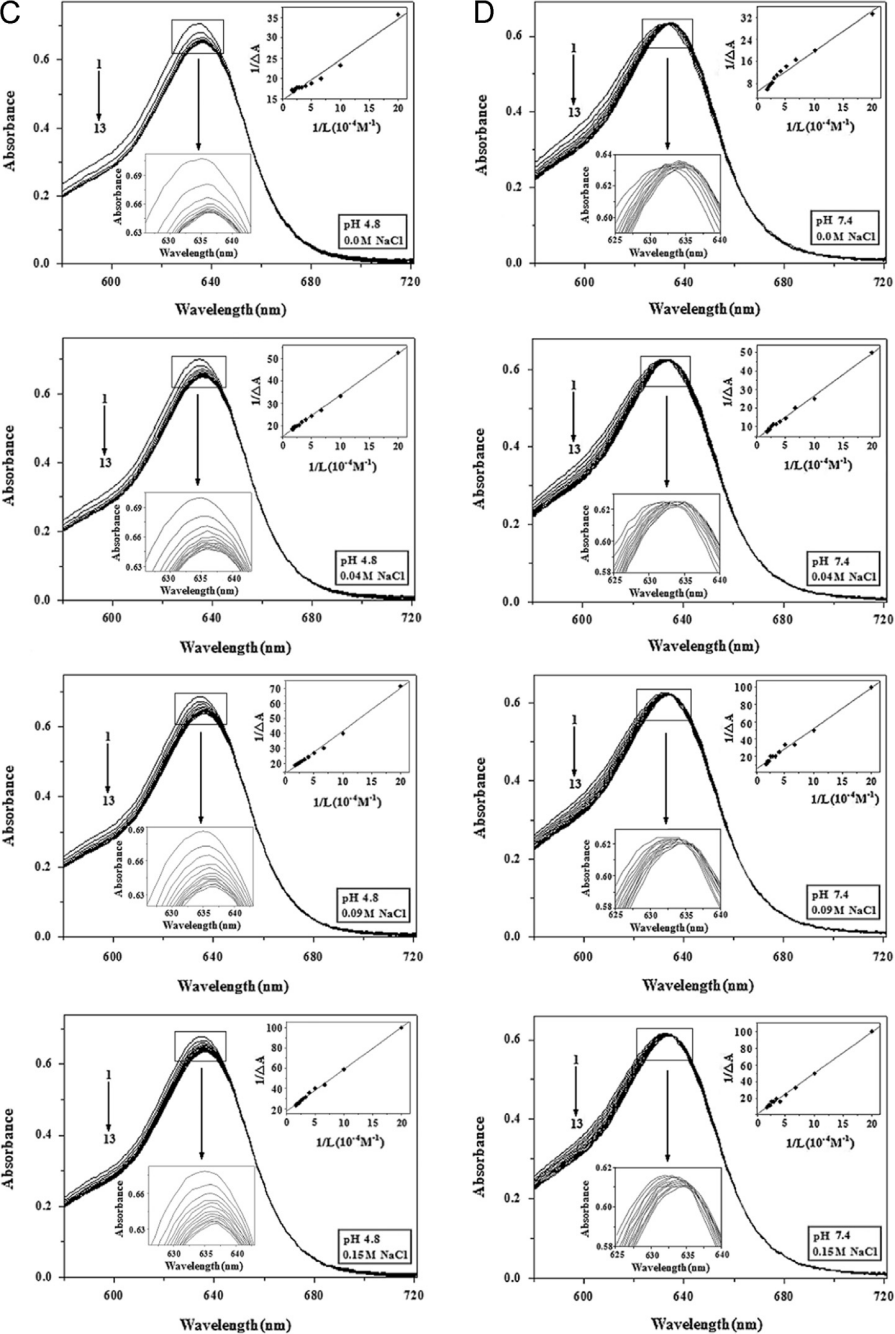
Absorption spectra of AR1 or AG50 with increasing the concentrations of BSA and Benesi–Hildebrand plots for the BSA-AR1 (A and B) or BSA-AG50 (C and D) complex in the presence of different salt concentrations at pH 4.8 and 7.4; *c*(AR1)=42 μM, *c*(AG50)=8 μM, *c*(BSA) 1–13=0, 5, 10, 15, 20, 25, 30, 35, 40, 45, 50, 55, 60 μM, *T*=298 °K.

The absorption spectra of AR1 (42 μM) or AG50 (8 μM) without or with NaCl concentration (0.04, 0.09 or 0.15 M) in the presence of BSA (0–60 μM) were recorded in the range of 300–600 nm by an UV-3600 spectrophotometer at pH 4.8 and 7.4, respectively; and corresponding absorption spectra were fitted using Origin 7.5 based on Eq. (2) (Fig. 9).

(2) $$\frac{1}{\Delta A}=\frac{1}{K_{B-H}\Delta A_{\max}}\times \frac{1}{\left[L\right]}+\frac{1}{\Delta A_{\max}}$$

#### 2.2.9 The effect of NaCl on the quenching plots of BSA-AR1/AG50 system

**Fig. 10:**
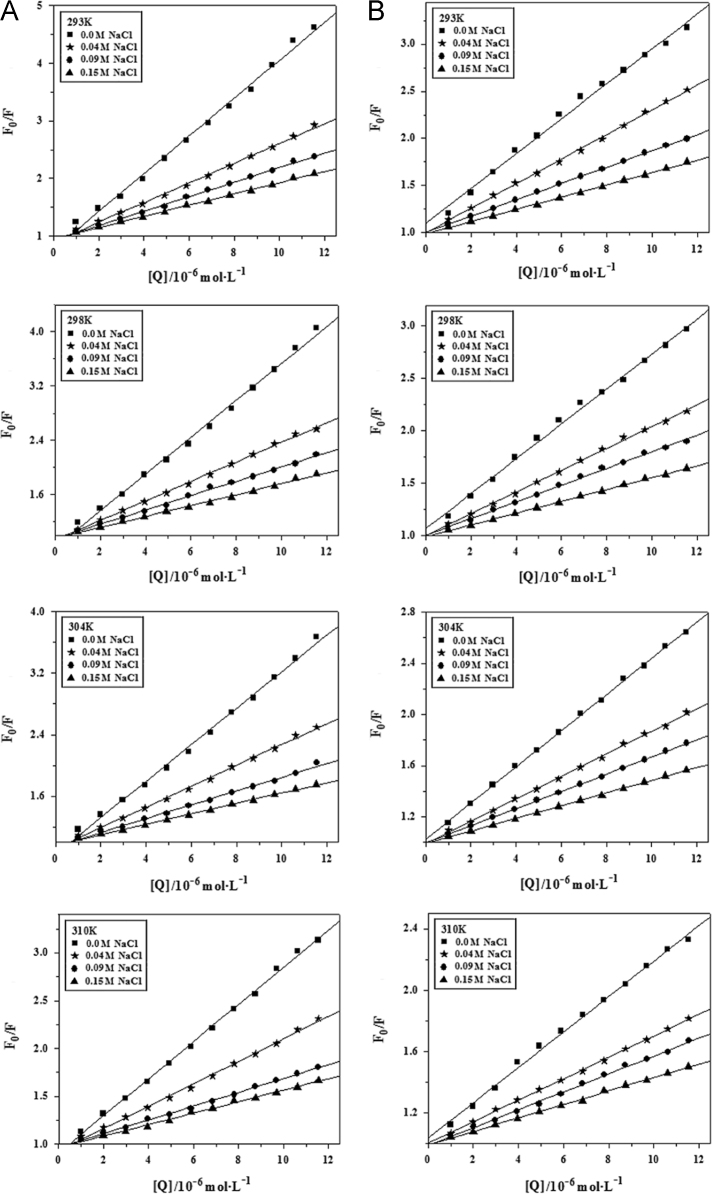

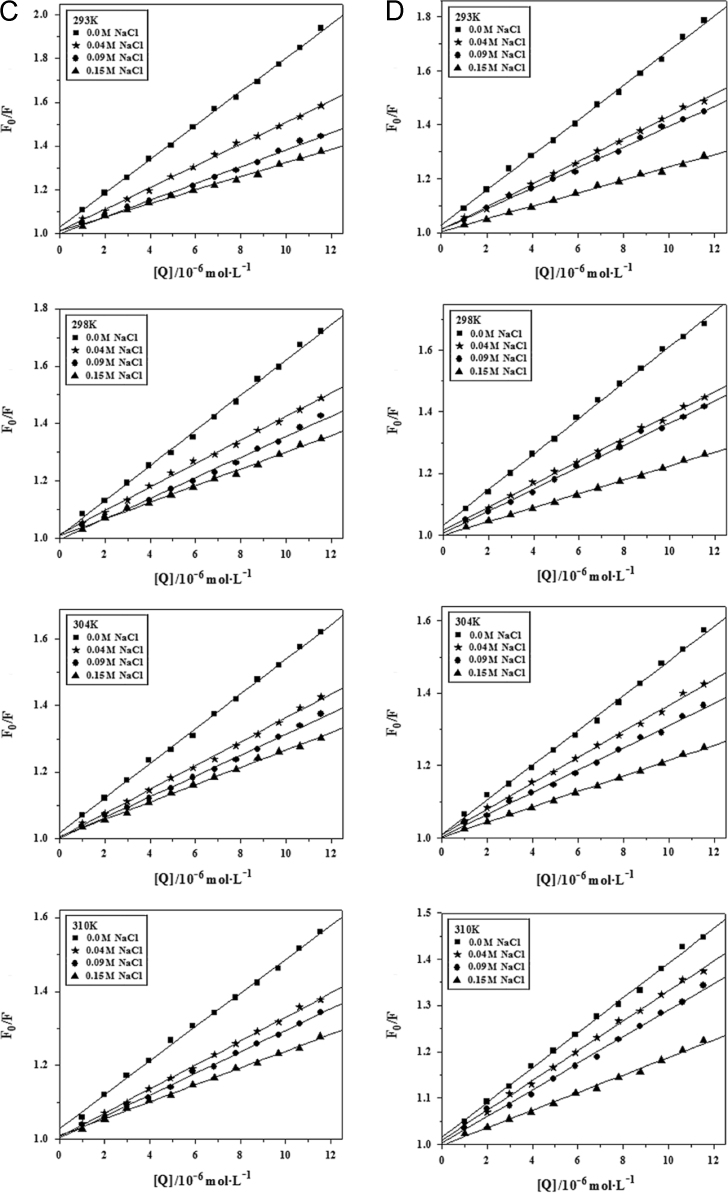
Effect of increasing NaCl concentrations on the quenching plots of BSA by AR1 (A and C) or AG50 (B and D) at pH 4.8 (A and B) or 7.4 (C and D), *λ*_ex_=280 nm.

The measured fluorescence quenching data of BSA by AR1/AG50 without or with NaCl (0.04, 0.09 or 0.15 M) at *T*=293, 298, 304 or 310 °K and pH=4.8 or 7.4 were corrected [1] and fitted by Origin 7.5 based on Eq. (1) (Fig. 10).

#### 2.2.10 The kinetics of BSA-AR1/AG50 system

**Fig. 11:**
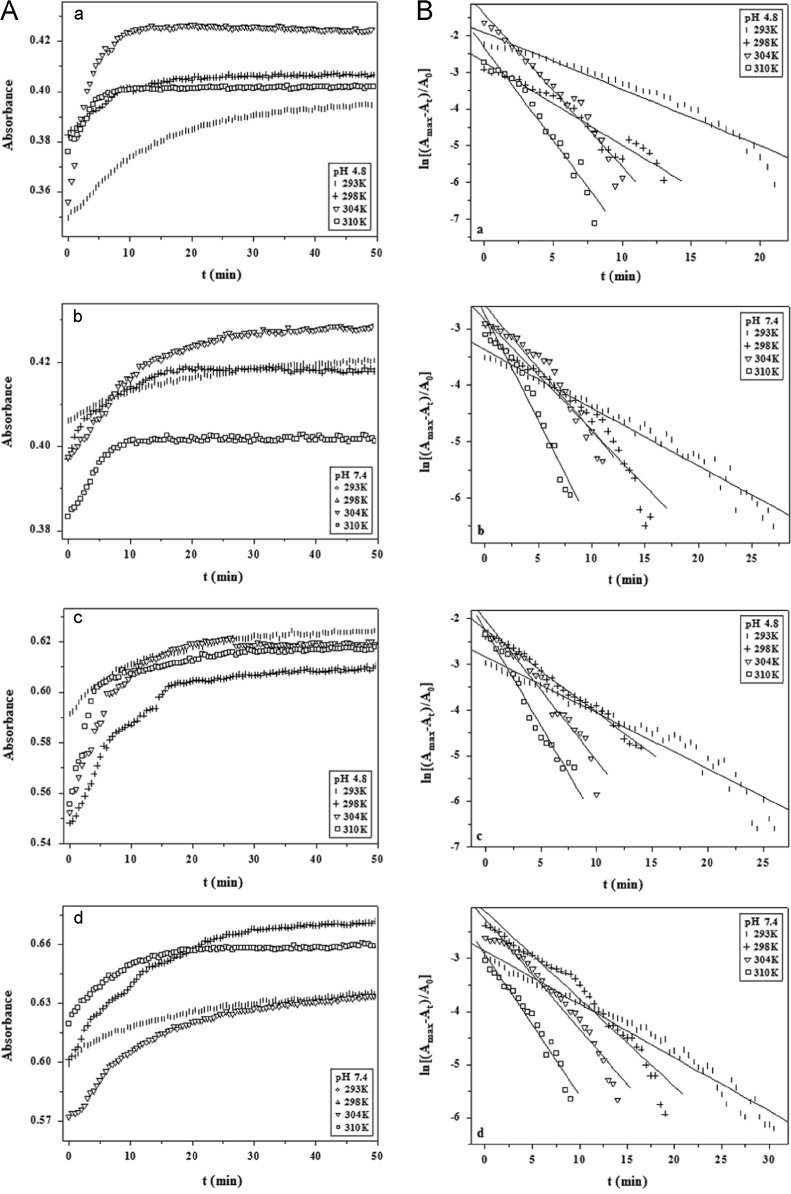
Plots (A) of BSA-AR1 (a and b) or BSA-AG50 (c and d) absorption *vs.* incubation time (*t*) and plots (B) of ln[(*A*_max_−*A*_t_)/*A*_0_] *vs*. incubation time (*t*) for the BSA-AR1 (a and b) or BSA-AG50 (c and d) system at different temperatures or pH. *c*(BSA)=2.0 μM, *c*(AR1)=*c*(AG50)=49.59 μM.

The absorption spectra of BSA-AR1/AG50 system (*T*=293, 298, 304 and 310 °K, pH=4.8 and 7.4) (Fig. 11A) were measured at 278 nm by an UV-3600 spectrophotometer at different time intervals. And semilogarithmic plots of ln[(*A*_max_−*A*_t_)/*A*_0_] *vs*. incubation time (*t*) for the BSA-AR1/AG50 system (Fig. 11B) were done by Origin 7.5.

#### 2.2.11 The UV-melting profiles of BSA-AR1/AG50 system

**Fig. 12:**
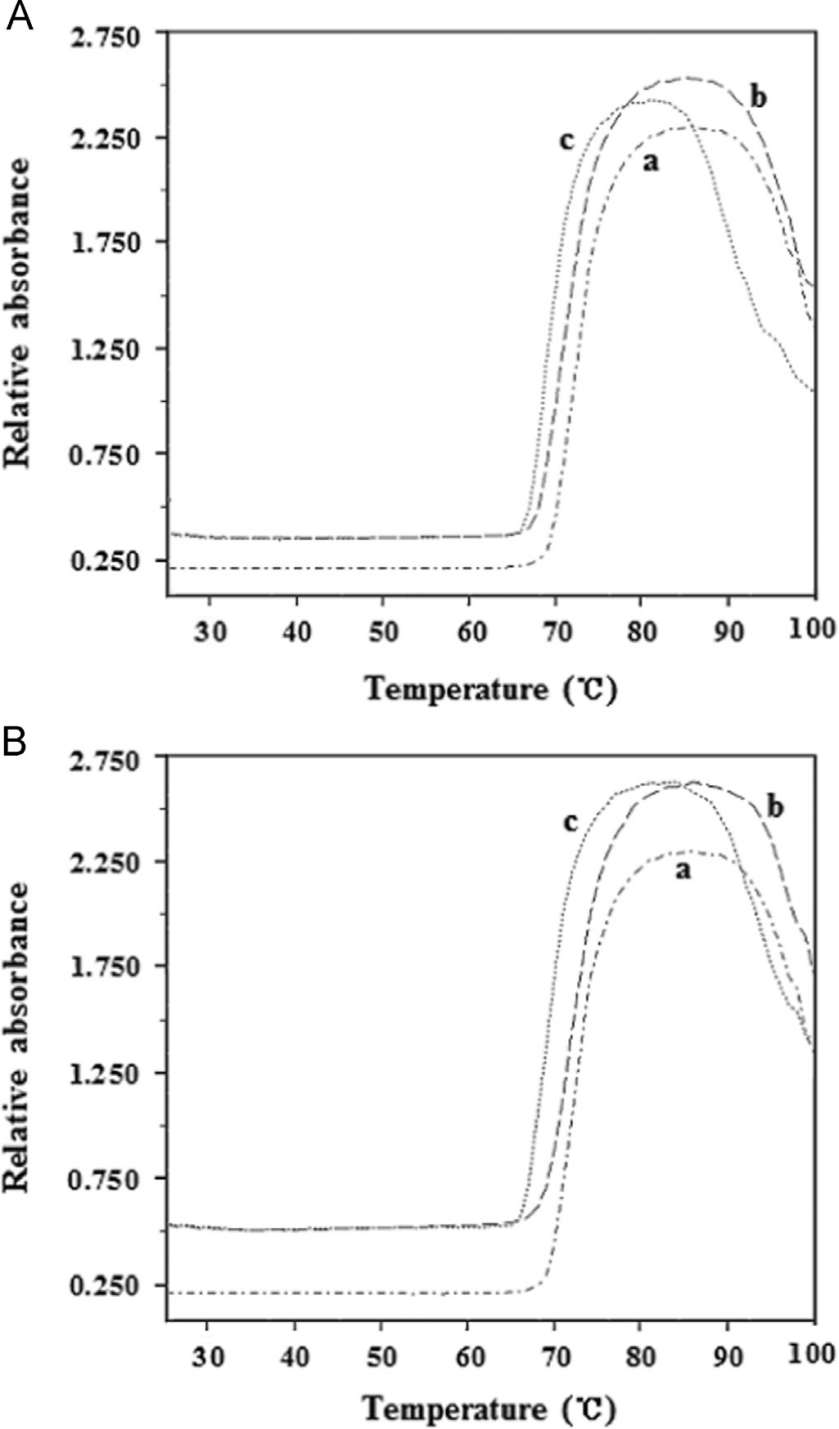
UV-melting profiles (absorbance change *vs.* temperature at 278 nm) of BSA and BSA-AR1 (A) or BSA-AG50 (B) system; curve a, b or c represents pattern for free BSA, BSA-AR1/AG50 or BSA-AR1/AG50 with NaCl (0.15 M). *c*(BSA)=5.0 μM, *c*(AR1)=*c*(AG50)=39.74 μM, pH=4.8.

Free BSA (5.0 μM) or BSA-AR1/AG50 (39.74 μM) complex without or with NaCl (0.15 M) were monitored at 278 nm by UV-3600 spectrophotometer with increasing temperature (from 25 to 100 °C with a rate of 1 °C/min). And their UV-melting profiles (Fig. 12) were fitted by MTLAB 2010.

#### 2.2.12 The synchronous fluorescence spectra

**Fig. 13:**
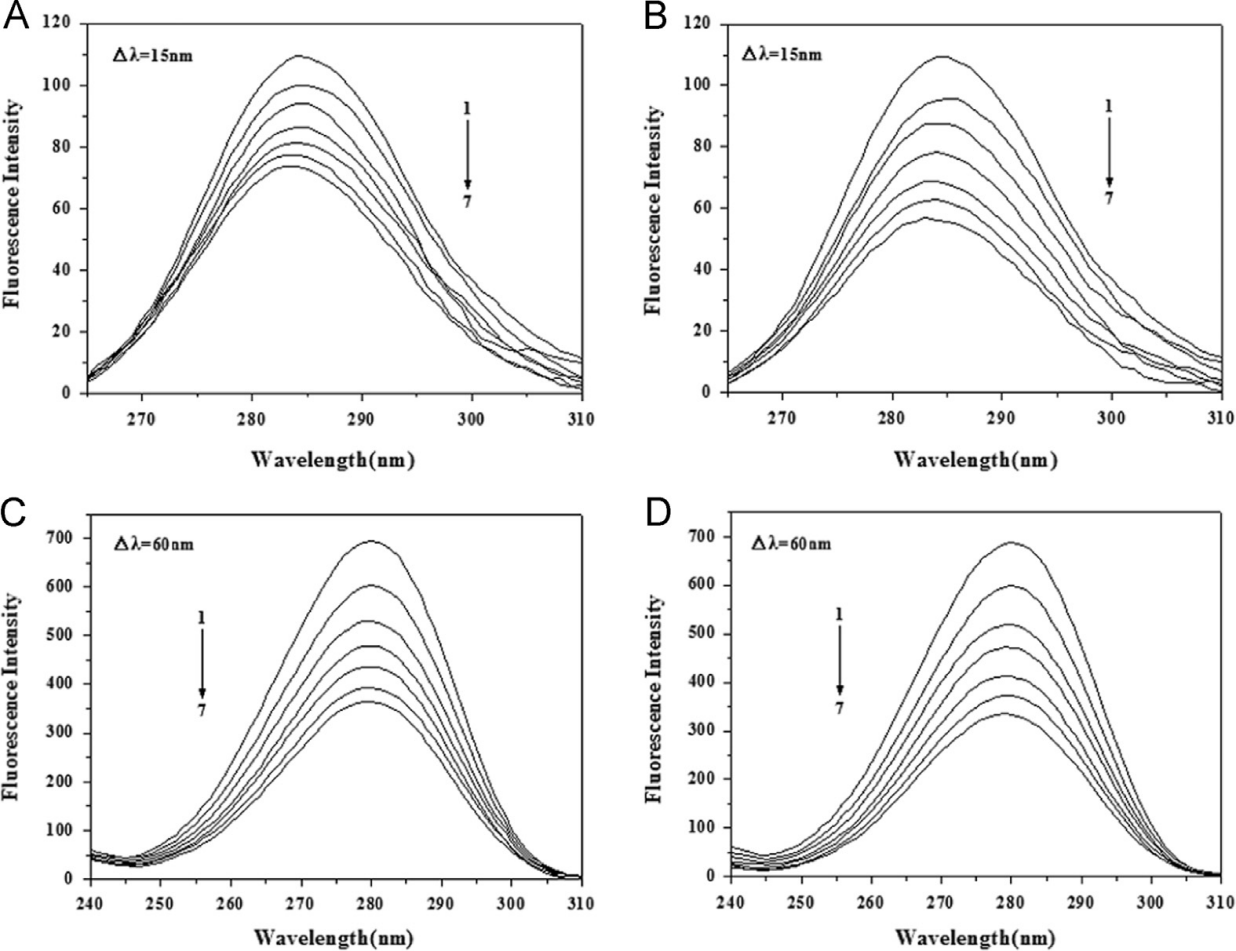
Synchronous fluorescence spectra of BSA-AR1 (A and C) or BSA-AG50 (B and D) system at Δ*λ*=15 and 60 nm; *c*(BSA)=2.0 μM, *c*(AR1)=*c*(AG50) 1–7=0, 1.99, 3.95, 5.88, 7.79, 9.68, 11.54 μM; *T*=298 °K.

Synchronous fluorescence spectra of BSA (2.0 μM) with the increasing AR1/AG50 concentration (0–75.00 μM) at Δ*λ*=15 and 60 nm were recorded using Cary Eclipse fluorescence spectrofluorimeter in the range of 250–500 nm. Other scanning parameters were the same as those of the fluorescence titration experiments. Besides, corresponding figures (Fig. 13) was made by Origin 7.5.

#### 2.2.13 FTIR spectra

**Fig. 14:**
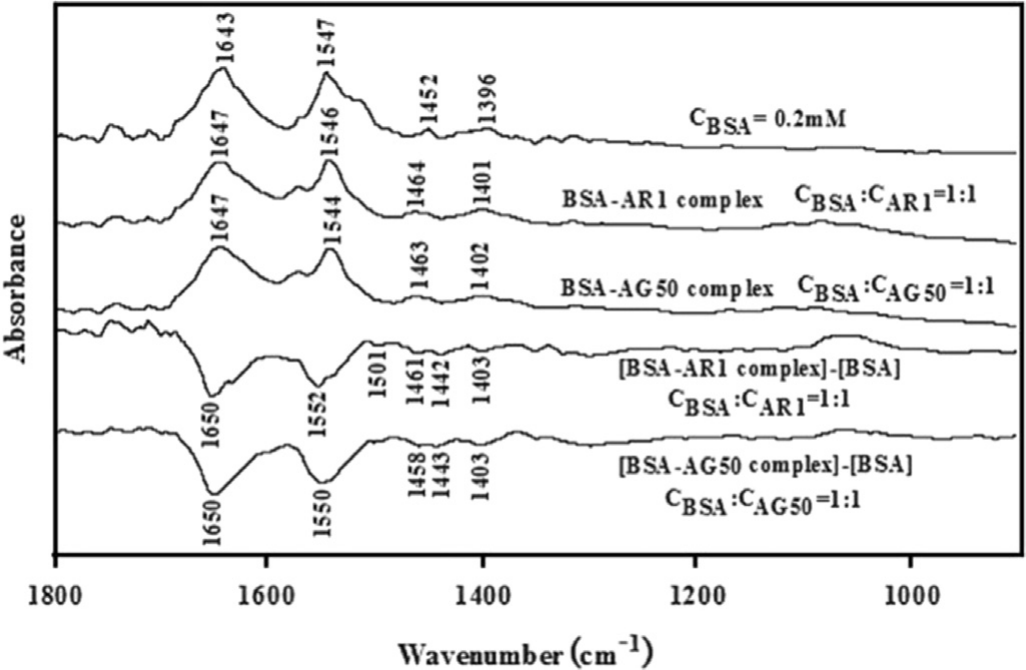
FTIR spectra in the 1800–900 cm^−1^ region for free BSA (0.2 mM), BSA-AR1 and BSA-AG50 complexes (the molar ratio of BSA to AR1 or AG50 is maintained at 1:1), and their corresponding difference spectra were indicated in the figure. The contribution of AR1 or AG50 is subtracted from the different spectra in this region.

FTIR spectra of free BSA (0.2 mM), BSA-AR1 and BSA-AG50 complexes (the molar ratio of BSA to AR1 or AG50 is maintained at 1:1) were recorded on Nicolet-6700 FTIR spectrometer *via* the attenuated total reflection (ATR) at a resolution of 4 cm^−1^ and 64 scans in the range of 400–4000 cm^−1^ at room temperature. The corresponding absorbance contributions of buffer and free AR1/AG50 solutions were recorded and digitally subtracted with the same instrumental parameters, and their FTIR spectra (Fig. 14) was done by OMNIC.

#### 2.2.14 The parameters of S–V plot

The parameters of fluorescence quenching for the BSA-AR1/AG50 system at different conditions were calculated using the S–V equation [1].

#### 2.2.15 Effect of pH, NaCl and ethanol on the binding parameters of BSA-AR1/AG50 system

The binding parameters of the two systems (Tables 2–4) were calculated using double logarithm regression curves, *Debye–Hückel* limiting law and *Van*’*t Hoff* equation based on the data of fluorescence quenching at different conditions, respectively [2], [3].

#### 2.2.16 The binding distances for the BSA-AR1/AG50system

According to Fig. 4, the binding distances of BSA-AR1/AG50 system at different conditions (Table 5) were calculated by Föster׳s non-radiative energy transfer theory [4].

#### 2.2.17 The values of *K*_B–H_ and Δ*G*_B–H_ for the BSA-AR1/AG50 system

According to Fig. 9, the values of *K*_B–H_ and Δ*G*_B–H_ for the BSA-AR1/AG50 complex (Table 6) were calculated using Eq. (2).

#### 2.2.18 Kinetics study of the BSA-AR1/AG50 system

The binding rate constants (*k*) for the BSA-AR1/AG50 system were calculated based on the data from Fig. 11 (Table 7).

## Figures and Tables

**Table 1:** The parameters of S–V plot for the BSA-AR1/AG50system at different conditions.

pH	NaCl (M)	*T* (K)	BSA-AR1				BSA-AG50			
			*K*_SV_ (×10^−4^ M^−1^)	*K*_q_ (×10^−12^ M^−1^ s^−1^)	*R*	*SD*	*K*_SV_ (×10^−4^ M^−1^)	*K*_q_ (×10^−12^ M^−1^ s^−1^)	*R*	*SD*
4.8	0	293	32.88	32.88	0.9973	0.0887	18.58	18.58	0.9969	0.0528
		298	27.17	27.17	0.9978	0.0655	16.68	16.68	0.9979	0.0393
		304	23.75	23.75	0.9981	0.0532	14.16	14.16	0.9997	0.0130
		310	19.43	19.43	0.9990	0.0322	11.56	11.56	0.9982	0.0249
									
	0.04	293	17.11	17.11	0.9988	0.0305	13.07	13.07	0.9995	0.0154
		298	14.37	14.37	0.9992	0.0206	10.41	10.41	0.9993	0.0139
		304	13.55	13.55	0.9988	0.0241	8.79	8.79	0.9993	0.0119
		310	11.74	11.74	0.9986	0.0229	7.00	7.00	0.9996	0.0070
									
	0.09	293	12.58	12.58	0.9990	0.0205	8.67	8.67	0.9999	0.0036
		298	10.52	10.52	0.9985	0.0209	7.97	7.97	0.9987	0.0147
		304	9.00	9.00	0.9975	0.0232	6.72	6.72	0.9998	0.0045
		310	7.19	7.19	0.9991	0.0112	5.85	5.85	0.9989	0.0098
									
	0.15	293	9.74	9.74	0.9990	0.0157	6.52	6.52	0.9993	0.0089
		298	8.02	8.02	0.9980	0.0185	5.66	5.66	0.9996	0.0062
		304	6.76	6.76	0.9990	0.0108	4.96	4.96	0.9996	0.0048
		310	5.97	5.97	0.9977	0.0148	4.41	4.41	0.9992	0.0063
										
7.4	0	293	7.76	7.76	0.9995	0.0086	6.46	6.46	0.9992	0.0097
		298	6.13	6.13	0.9988	0.0111	5.81	5.81	0.9992	0.0084
		304	5.22	5.22	0.9993	0.0073	4.79	4.79	0.9985	0.0095
		310	4.59	4.59	0.9987	0.0084	3.77	3.77	0.9990	0.0062
									
	0.04	293	5.01	5.01	0.9972	0.0136	4.20	4.20	0.9995	0.0051
		298	3.76	3.76	0.9955	0.0130	3.75	3.75	0.9992	0.0055
		304	3.59	3.59	0.9990	0.0058	3.58	3.58	0.9989	0.0061
		310	3.25	3.25	0.9995	0.0038	3.22	3.22	0.9992	0.0046
									
	0.09	293	3.84	3.84	0.9976	0.0096	3.81	3.81	0.9984	0.0077
		298	3.59	3.59	0.9969	0.0103	3.58	3.58	0.9979	0.0084
		304	3.14	3.14	0.9982	0.0068	3.07	3.07	0.9981	0.0068
		310	2.91	2.91	0.9985	0.0058	2.87	2.87	0.9971	0.0080
									
	0.15	293	3.10	3.10	0.9976	0.0078	2.39	2.39	0.9984	0.0050
		298	2.90	2.90	0.9981	0.0066	2.25	2.25	0.9993	0.0030
		304	2.60	2.60	0.9989	0.0044	2.13	2.13	0.9994	0.0027
		310	2.29	2.29	0.9986	0.0044	1.90	1.90	0.9976	0.0048

*R* and *SD* are the correlation coefficient and the standard deviation for the S–V plots, respectively.

**Table 2:** Effect of pH on the parameters calculated by *Debye–Hückel* limiting law for the BSA-AR1/AG50 system.

Ligind	*T* (K)	*I*^1/2^	pH 4.8						*I*^1/2^	pH 7.4						Δ*Z*_P_=*Z*_P,pH4.8_-*Z*_P,pH7.4_
			log(*K*_exp.1_)	log(*K*_exp.2_)	log(*K*_exp.3_)	log(*K*)	*K*_eq_ (×10^−3^ M^−1^)	*Z*_P_		log(*K*_exp.1_)	log(*K*_exp.2_)	log(*K*_exp.3_)	log(*K*)	*K*_eq_ (×10^−3^ M^−1^)	*Z*_P_	

AR1	293	0.22	6.21401	6.00249	6.08425	6.10025	4378.9	1.18	0.26	4.45338	4.25311	4.32311	4.34320	33.6	0.35	0.83
		0.30	6.01412	5.79428	5.90369	5.90403			0.33	4.38801	4.18799	4.25857	4.27819			
		0.37	5.83139	5.61051	5.73388	5.72526			0.40	4.35407	4.15423	4.22478	4.24436			
		0.45	5.64213	5.42627	5.53885	5.53575			0.47	4.29632	4.09638	4.16689	4.18653			
	298	0.22	6.15317	5.93821	6.04122	6.04420	4210.2	1.30	0.26	4.38491	4.18479	4.25536	4.27502	30.9	0.41	0.89
		0.30	5.95846	5.73241	5.84623	5.84570			0.33	4.32073	4.12087	4.19098	4.21086			
		0.37	5.72893	5.52828	5.62019	5.62580			0.40	4.29111	4.09109	4.16146	4.18122			
		0.45	5.55324	5.33887	5.45339	5.44850			0.47	4.20272	4.00248	4.07338	4.09286			
	304	0.22	6.09431	5.87959	5.97375	5.98255	4023.8	1.42	0.26	4.30889	4.09871	4.18949	4.19903	25.6	0.40	1.02
		0.30	5.89338	5.67842	5.78608	5.78596			0.33	4.25238	4.05222	4.12254	4.14238			
		0.37	5.64212	5.42808	5.54008	5.53676			0.40	4.22402	4.02478	4.09677	4.11519			
		0.45	5.46171	5.24669	5.35129	5.35323			0.47	4.13521	3.93449	4.00350	4.02440			
	310	0.22	6.02188	5.80111	5.92981	5.91760	3578.4	1.50	0.26	4.24029	4.04021	4.10992	4.13014	22.8	0.44	1.06
		0.30	5.83412	5.61538	5.73792	5.72914			0.33	4.20342	4.00328	4.07386	4.09352			
		0.37	5.55041	5.36749	5.41161	5.44317			0.40	4.13913	3.93847	4.00851	4.02870			
		0.45	5.38409	5.16861	5.30047	5.28439			0.47	4.06603	3.86537	3.93603	3.95581			
AG50	293	0.22	5.17015	4.97229	5.04515	5.06253	162.1	0.66	0.26	4.32163	4.12127	4.19274	4.21188	20.2	0.34	0.32
		0.30	5.10342	4.91228	4.99252	5.00274			0.33	4.29691	4.09679	4.16763	4.18711			
		0.37	5.06033	4.85987	4.93082	4.95034			0.40	4.27288	4.07312	4.14321	4.16307			
		0.45	5.01017	4.81032	4.89901	4.90650			0.47	4.24649	4.04681	4.11746	4.13692			
	298	0.22	5.13221	4.93139	5.01515	5.02625	154.6	0.74	0.26	4.27812	4.07788	4.14908	4.16836	18.8	0.42	0.32
		0.30	5.06133	4.86007	4.95539	4.95893			0.33	4.23901	4.03919	4.10982	4.12934			
		0.37	5.02049	4.82311	4.89861	4.91407			0.40	4.20907	4.00913	4.07949	4.09923			
		0.45	4.96028	4.75232	4.84058	4.85106			0.47	4.18849	3.98841	4.05953	4.07881			
	304	0.22	5.09308	4.89562	4.96882	4.98584	139.0	0.74	0.26	4.23508	4.02812	4.06773	4.11031	16.2	0.41	0.33
		0.30	5.02351	4.82609	4.90182	4.91714			0.33	4.17893	3.97827	4.05037	4.06919			
		0.37	4.98413	4.78257	4.85455	4.87375			0.40	4.12771	3.93549	4.02959	4.03093			
		0.45	4.92152	4.72447	4.79961	4.81520			0.47	4.12221	3.93048	4.02951	4.02740			
	310	0.22	5.05412	4.85208	4.92463	4.94361	128.9	0.79	0.26	4.19188	3.99182	4.06278	4.08216	16.9	0.62	0.17
		0.30	4.98051	4.78229	4.86229	4.87503			0.33	4.13839	3.93851	4.00956	4.02882			
		0.37	4.94652	4.74648	4.82031	4.83777			0.40	4.08361	3.88339	3.95458	3.97386			
		0.45	4.87117	4.67391	4.74542	4.76350			0.47	4.07203	3.87187	3.94297	3.96229			

Ligind	*T* (K)	*I*^1/2^	pH 4.8					*I*^1/2^	pH 7.4				
			$\Delta G_{\exp.1}^{0}$ (kJ mol^−1^)	$\Delta G_{\exp.2}^{0}$ (kJ mol^−1^)	$\Delta G_{\exp.3}^{0}$ (kJ mol^−1^)	Δ*G*° (kJ mol^−1^)	$\Delta G_{I\to 0}^{0}$ (kJ mol^−1^)		$\Delta G_{\exp.1}^{0}$ (kJ mol^−1^)	$\Delta G_{\exp.2}^{0}$ (kJ mol^−1^)	$\Delta G_{\exp.3}^{0}$ (kJ mol^−1^)	Δ*G*° (kJ mol^−1^)	$\Delta G_{I\to 0}^{0}$ (kJ mol^−1^)

AR1	293	0.22	−34.9	−33.7	−34.1	−34.2	−37.3	0.26	−25.0	−23.7	−24.2	−24.3	−25.4
		0.30	−33.7	−32.5	−33.1	−33.1		0.33	−24.6	−23.5	−23.9	−24.0	
		0.37	−32.7	−31.4	−32.1	−32.1		0.40	−24.4	−23.3	−23.7	−23.8	
		0.45	−31.6	−30.4	−31.0	−31.0		0.47	−23.8	−23.3	−23.2	−23.3	
	298	0.22	−35.1	−33.9	−34.4	−34.5	−37.8	0.26	−25.0	−23.9	−24.3	−24.4	−25.6
		0.30	−34.0	−32.7	−33.4	−33.4		0.33	−24.7	−23.5	−23.9	−24.0	
		0.37	−32.7	−31.5	−32.1	−32.1		0.40	−24.5	−23.3	−23.7	−23.8	
		0.45	−31.7	−30.5	−31.1	−31.1		0.47	−24.0	−23.3	−23.2	−23.4	
	304	0.22	−35.4	−34.2	−34.8	−34.8	−38.4	0.26	−25.1	−23.9	−24.3	−24.4	−25.7
		0.30	−34.3	−33.0	−33.7	−33.7		0.33	−24.8	−23.6	−24.0	−24.2	
		0.37	−32.8	−31.7	−32.1	−32.2		0.40	−24.5	−23.4	−23.8	−23.9	
		0.45	−31.8	−30.6	−31.3	−31.2		0.47	−24.0	−23.3	−23.2	−23.4	
	310	0.22	−35.8	−34.4	−35.2	−35.1	−39.0	0.26	−25.2	−23.9	−24.4	−24.5	−25.9
		0.30	−34.6	−33.3	−34.0	−34.0		0.33	−24.9	−23.7	−24.1	−24.3	
		0.37	−32.9	−31.8	−32.2	−32.3		0.40	−24.6	−23.4	−23.8	−23.9	
		0.45	−31.9	−30.6	−31.4	−31.3		0.47	−24.0	−23.3	−23.2	−23.4	
AG50	293	0.22	−29.0	−27.9	−28.3	−28.4	−29.2	0.22	−24.2	−23.1	−23.5	−23.6	−24.1
		0.30	−28.6	−27.5	−28.0	−28.0		0.30	−24.1	−23.0	−23.3	−23.5	
		0.37	−28.4	−27.3	−27.7	−27.8		0.37	−23.9	−22.8	−23.2	−23.3	
		0.45	−28.1	−27.0	−27.5	−27.5		0.45	−23.8	−22.7	−23.1	−23.2	
	298	0.22	−29.3	−28.2	−28.6	−28.7	−29.6	0.22	−24.4	−23.3	−23.6	−23.8	−24.4
		0.30	−28.9	−27.8	−28.3	−28.3		0.30	−24.2	−23.1	−23.5	−23.6	
		0.37	−28.7	−27.5	−27.9	−28.0		0.37	−24.0	−22.9	−23.3	−23.4	
		0.45	−28.3	−27.2	−27.7	−27.7		0.45	−23.9	−22.8	−23.2	−23.3	
	304	0.22	−29.6	−28.5	−28.9	−29.0	−30.0	0.22	−24.6	−23.5	−23.8	−24.0	−24.7
		0.30	−29.2	−28.1	−28.5	−28.6		0.30	−24.4	−23.2	−23.6	−23.7	
		0.37	−29.0	−27.8	−28.3	−28.4		0.37	−24.1	−22.9	−23.4	−23.5	
		0.45	−28.6	−27.5	−27.9	−28.0		0.45	−24.0	−22.8	−23.2	−23.3	
	310	0.22	−30.0	−28.8	−29.2	−29.3	−30.3	0.22	−24.9	−23.7	−24.0	−24.2	−25.0
		0.30	−29.6	−28.4	−28.8	−28.9		0.30	−24.5	−23.3	−23.8	−23.9	
		0.37	−29.3	−28.2	−28.6	−28.7		0.37	−24.2	−23.0	−23.5	−23.6	
		0.45	−28.9	−27.7	−28.2	−28.3		0.45	−24.1	−23.0	−23.5	−23.5	

*K*_exp1_, *K*_exp2_ and *K*_exp3_ denote the binding constants of BSA-AR1/AG50 system in triplicate, respectively; corresponding change of free energy (Δ*G*°) is $\Delta G_{\exp.1}^{0}$, $\Delta G_{\exp.2}^{0}$ and $\Delta G_{\exp.3}^{0}$; log (*K*) and Δ*G*° express the average values of log (*K*_exp1_), log (*K*_exp2_) and log (*K*_exp3_), and $\Delta G_{\exp.1}^{0}$, $\Delta G_{\exp.2}^{0}$ and $\Delta G_{\exp.3}^{0}$, respectively.

**Table 3:** The binding constants *K*, binding sites number *n* and thermodynamic parameters for the BSA-AR1/AG50 system at different conditions.

Systems	NaCl (M)	*T* (K)	pH4.8							pH 7.4						
			*K* (×10^−3^ M^−1^)	*n*	*R*^a^	Δ*G*° (kJ mol^−1^)	Δ*H*° (kJ mol^−1^)	Δ*S*° (J mol^−1^ K^−1^)	*R*^b^	*K* (×10^−3^ M^−1^)	*n*	*R*^a^	Δ*G*° (kJ mol^−1^)	Δ*H*° (kJ mol^−1^)	Δ*S*° (J mol^−1^ K^−1^)	*R*^b^
BSA-AR1	0	293	1259.65	1.13	0.9981	−34.22	−18.59	53.34	0.9998	22.04	0.89	0.9990	−24.35	−21.80	8.71	0.9998
		298	1107.06	1.13	0.9995	−34.48				18.84	0.90	0.9958	−24.40			
		304	960.62	1.13	0.9997	−34.80				15.81	0.89	0.9987	−24.45			
		310	827.18	1.13	0.9994	−35.12				13.49	0.89	0.9991	−24.50			
	0.04	293	801.73	1.14	0.9999	−33.10	−17.81	52.21	0.9995	18.98	0.92	0.9969	−23.96	−18.95	17.11	0.9959
		298	700.91	1.14	0.9991	−33.36				16.25	0.92	0.9989	−24.05			
		304	610.89	1.14	0.9998	−33.68				13.88	0.92	0.9975	−24.15			
		310	535.97	1.14	0.9998	−33.99				12.40	0.92	0.9980	−24.25			
	0.09	293	531.20	1.13	0.9996	−32.08	−28.48	12.29	0.9987	17.55	0.93	0.9973	−23.82	−21.75	7.08	0.9978
		298	422.46	1.12	0.9992	−32.14				15.18	0.93	0.9946	−23.86			
		304	344.16	1.12	0.9991	−32.22				13.04	0.93	0.9975	−23.90			
		310	277.44	1.12	0.9992	−32.29				10.68	0.91	0.9944	−23.94			
	0.15	293	343.36	1.11	0.9997	−31.02	−25.87	17.56	0.9972	15.36	0.93	0.9966	−23.42	−23.12	1.03	0.9940
		298	280.86	1.11	0.9994	−31.10				12.38	0.92	0.9976	−23.43			
		304	225.54	1.11	0.9996	−31.21				10.58	0.92	0.9968	−23.44			
		310	192.48	1.11	0.9967	−31.32				9.03	0.92	0.9990	−23.44			
BSA- AG50	0	293	115.49	0.95	0.9985	−28.40	−12.11	55.58	0.9999	16.29	0.88	0.9990	−23.61	−13.63	34.06	0.9919
		298	106.23	0.96	0.9990	−28.68				14.74	0.88	0.9990	−23.78			
		304	96.79	0.97	0.9999	−29.01				12.89	0.89	0.9966	−23.98			
		310	87.82	0.97	0.9988	−29.34				12.08	0.90	0.9994	−24.19			
	0.04	293	100.63	0.98	0.9996	−28.05	−12.94	51.55	0.9992	15.39	0.91	0.9977	−23.46	−16.29	24.47	0.9952
		298	90.98	0.99	0.9995	−28.31				13.47	0.91	0.9996	−23.58			
		304	82.63	1.00	0.9982	−28.62				11.73	0.90	0.9975	−23.73			
		310	74.99	1.00	0.9992	−28.93				10.69	0.90	0.9974	−23.87			
	0.09	293	89.19	1.00	0.9999	−27.76	−11.52	55.42	0.9997	14.56	0.91	0.9986	−23.33	−19.38	13.51	0.9989
		298	82.05	1.00	0.9989	−28.04				12.57	0.91	0.9945	−23.40			
		304	74.77	1.01	0.9999	−28.37				10.74	0.91	0.9950	−23.48			
		310	68.83	1.02	0.9988	−28.70				9.42	0.90	0.9928	−23.56			
	0.15	293	80.63	1.02	0.9994	−27.50	−14.14	45.59	0.9947	13.71	0.95	0.9982	−23.20	−17.53	19.33	0.9985
		298	70.97	1.02	0.9986	−27.72				11.99	0.95	0.9983	−23.29			
		304	65.34	1.03	0.9995	−28.00				10.65	0.94	0.9982	−23.41			
		310	58.01	1.03	0.9992	−28.27				9.17	0.94	0.9957	−23.53			

*R*^a^ and *R*^b^ are the correlation coefficients for *K* values and *Van’t Hoff* plots, respectively.

**Table 4:** Effects of ethanol or pH on the binding parameters of BSA-AR1/AG50 system.

Systems	ethanol (v/v)	parameters	pH			
			4.8	5.5	6.3	7.4
BSA-AR1	0	*K* (×10^−3^ M^−1^) (×10^−3^ L mol^−1^)	1107.06	169.29	59.65	18.84
		*n*	1.13	1.03	0.97	0.90
		*R*	0.9995	0.9996	0.9987	0.9958

	5	*K* (×10^−3^ M^−1^) (×10^−3^ L mol^−1^)	553.22	42.99	36.33	14.16
		*n*	1.13	0.96	0.98	0.89
		*R*	0.9997	0.9984	0.9998	0.9964

	10	*K* (×10^−3^ M^−1^) (×10^−3^ L mol^−1^)	169.53	27.72	25.20	10.88
		*n*	1.07	0.97	0.99	0.90
		*R*	0.9997	0.9978	0.9954	0.9931

BSA-AG50	0	*K* (×10^−3^ M^−1^) (×10^−3^ L mol^−1^)	106.23	84.31	51.00	14.74
		*n*	0.96	0.95	0.94	0.88
		*R*	0.9990	0.9993	0.9990	0.9990

	5	*K* (×10^−3^ M^−1^) (×10^−3^ L mol^−1^)	57.08	36.20	26.76	7.25
		*n*	0.98	0.98	0.96	0.89
		*R*	0.9991	0.9966	0.9945	0.9997

	10	*K* (×10^−3^ M^−1^) (×10^−3^ L mol^−1^)	32.95	20.11	16.61	5.59
		*n*	1.01	0.99	1.01	0.90
		*R*	0.9995	0.9952	0.9981	0.9954

**Table 5:** The binding distances for the BSA-AR1/AG50 system at different pH and salt concentrations.

Systems	pH	*r* (nm)			
		0.0 M NaCl	0.04 M NaCl	0.09 M NaCl	0.15 M NaCl
BSA-AR1	4.8	2.62	2.84	3.05	3.18
	5.5	2.99	3.07	3.42	3.55
	6.3	3.19	3.66	3.72	3.85
	7.4	3.38	3.72	3.75	3.91
					
BSA-AG50	4.8	2.91	3.17	3.42	3.55
	5.5	3.01	3.23	3.46	3.57
	6.3	3.21	3.68	3.76	3.88
	7.4	3.45	3.74	3.80	3.92

**Table 6:** The values of *K*_B–H_ and Δ*G*_B–H_ for the BSA-AR1/AG50 complex at different conditions.

Systems	pH	Parameters	NaCl (M)			
			0	0.04	0.09	0.15
BSA-AR1	4.8	*K*_B–H_ (×10^−3^ L mol^−1^)	158.62	139.37	77.02	65.45
		*R*	0.9866	0.9962	0.9963	0.9975
		Δ*G*_B__–__H_ (kJ mol^−1^)	−29.67	−29.35	−27.88	−10.35

	7.4	*K*_B__–__H_ (×10^−3^ L mol^−1^)	45.00	38.05	35.20	23.28
		*R*	0.9999	0.9993	0.9983	0.9994
		Δ*G*_B__–H_ (kJ mol^−1^)	−9.43	−9.02	−8.82	−7.80

BSA-AG50	4.8	*K*_B__–H_ (×10^−3^ L mol^−1^)	148.48	82.99	47.08	41.65
		*R*	0.9871	0.9998	0.9978	0.9989
		Δ*G*_B__–H_ (kJ mol^−1^)	−12.39	−10.95	−9.54	−9.24

	7.4	*K*_B__–H_ (×10^−3^ L mol^−1^)	36.12	15.24	10.54	3.94
		*R*	0.9870	0.9980	0.9954	0.9974
		Δ*G*_B__–H_ (kJ mol^−1^)	−8.89	−6.75	−5.84	−3.40

**Table 7:** The binding rate constants *k* and corresponding statistical parameters for the BSA-AR1/AG50 system at different conditions.

Systems	NaCl (M)	*T* (K)	pH 4.8						pH 7.4					
			*k*_exp.1_ (×10^3^ min^−1^)	*k*_exp.2_ (×10^3^ min^−1^)	*k*_exp.3_ (×10^3^ min^−1^)	*k* (×10^3^ min^−1^)	*R*	*SD*	*k*_exp.1_ (×10^3^ min^−1^)	*k*_exp.2_ (×10^3^ min^−1^)	*k*_exp.3_ (×10^3^ min^−1^)	*k* (×10^3^ min^−1^)	*R*	*SD*
BSA-AR1	0	293	150.4	157.9	154.3	154.2	0.9708	0.2423	99.6	106.8	102.6	103.0	0.9858	0.1416
		298	223.9	227.4	229.4	226.9	0.9699	0.2307	194.2	198.4	195.1	195.9	0.9658	0.2509
		304	410.7	418.6	418.4	415.9	0.9804	0.2658	218.4	225.8	221.2	221.8	0.9643	0.2116
		310	506.4	513.2	508.9	509.5	0.9733	0.3134	374.2	379.9	377.5	377.2	0.9746	0.2262
	0.15	298	179.9	188.2	189.5	185.9	0.9651	0.1897	168.9	173.5	175.4	172.6	0.9666	0.1919
														
BSA-AG50	0	293	117.9	125.1	124.2	122.4	0.9691	0.2429	96.7	105.8	98.7	100.4	0.9848	0.1613
		298	178.2	184.6	180.2	181.0	0.9939	0.0869	160.8	164.1	168.3	164.4	0.9745	0.2186
		304	305.1	311.2	305.9	307.4	0.9704	0.2435	205.1	210.6	205.3	207.0	0.9780	0.1914
		310	414.8	425.3	414.2	418.1	0.9840	0.1973	261.4	269.8	265.9	265.7	0.9782	0.1632
	0.15	298	158.1	166.3	168.8	164.4	0.9866	0.1817	124.3	134.6	126.9	128. 6	0.9598	0.2607

*R* and *SD* are the correlation coefficient and the standard deviation for *k* values, respectively; *k*_exp.1,_*k*_exp.2_ and *k*_exp.3_ denote the binding rate constants of the BSA-AR1/AG50 system in triplicate, respectively; *k* is the average values of *k*_exp.1,_*k*_exp.2_ and *k*_exp.3._
